# Energy Intake of Men With Excess Weight During Normobaric Hypoxic Confinement

**DOI:** 10.3389/fphys.2021.801833

**Published:** 2022-01-12

**Authors:** Igor B. Mekjavic, Mojca Amon, Elizabeth J. Simpson, Roger Kölegård, Ola Eiken, Ian A. Macdonald

**Affiliations:** ^1^Department of Automation, Biocybernetics and Robotics, Jozef Stefan Institute, Ljubljana, Slovenia; ^2^Metabolic and Molecular Physiology Group, Faculty of Medicine and Health Sciences University of Nottingham Queen’s Medical Centre, Nottingham, United Kingdom; ^3^Division of Environmental Physiology, School of Chemistry, Biotechnology and Health, KTH Royal Institute of Technology, Stockholm, Sweden

**Keywords:** obesity, hypoxia, altitude, weight loss, metabolism

## Abstract

Due to the observations of weight loss at high altitude, normobaric hypoxia has been considered as a method of weight loss in obese individuals. With this regard, the aim of the present study was to determine the effect of hypoxia *per se* on metabolism in men with excess weight. Eight men living with excess weight (125.0 ± 17.7 kg; 30.5 ± 11.1 years, BMI: 37.6 ± 6.2 kg⋅m^–2^) participated in a randomized cross-over study comprising two 10-day confinements: normobaric (altitude of facility ≃ 940 m) normoxia (NORMOXIA; P_*I*_O_2_ = 133 mmHg), and normobaric hypoxia (HYPOXIA). The P_*I*_O_2_ in the latter was reduced from 105 (simulated altitude of 2,800 m) to 98 mmHg (simulated altitude of 3,400 m over 10 days. Before, and at the end of each confinement, participants completed a meal tolerance test (MTT). Resting energy expenditure (REE), circulating glucose, GLP-1, insulin, catecholamines, ghrelin, peptide-YY (PYY), leptin, gastro-intestinal blood flow, and appetite sensations were measured in fasted and postprandial states. Fasting REE increased after HYPOXIA (+358.0 ± 49.3 kcal⋅day^–1^, *p* = 0.03), but not after NORMOXIA (−33.1 ± 17.6 kcal⋅day^–1^). Postprandial REE was also significantly increased after HYPOXIA (*p* ≤ 0.05), as was the level of PYY. Furthermore, a tendency for decreased energy intake was concomitant with a significant body weight reduction after HYPOXIA (−0.7 ± 0.2 kg) compared to NORMOXIA (+1.0 ± 0.2 kg). The HYPOXIA trial increased the metabolic requirements, with a tendency toward decreased energy intake concomitant with increased PYY levels supporting the notion of a hypoxia-induced appetite inhibition, that could potentially lead to body weight reduction. The greater postprandial blood-glucose response following hypoxic confinement, suggests the potential development of insulin resistance.

## New Findings

- Prolongued exposure of humans to high altitude has been observed to cause weight loss, and the etiology of this hypoxia-induced loss of body mass remains relatively unresolved. Previous observations of this high altitude anorexia phenomenon in climbers may have been confounded by increased physical activity, insufficient food availability and environmental cold.
- A controlled 10-day normobaric hypoxic confinement of men with excess weight revealed a significant appetite suppression with a concomitant loss of body mass when compared to similar confinement in normobaric normoxia.

## Introduction

Obesity has become a global epidemic. Considering the morbidity, mortality, social and economic burdens related to this malady, it is obvious that effective prevention strategies and treatments are needed. Once body mass index (BMI) exceeds 30 kg.m^–2^, mortality rate from all causes, especially cardiovascular disease, increases by 50–100% (Poirier and Després, 2001). In addition to reduced energy intake, the beneficial effects of physical activity in preventing or alleviating chronic diseases and increasing physiological and psychological well-being are well documented (Hudon et al., 2008). However, since obesity also increases cardiovascular risk, the prescription of exercise must be conducted with care. Particularly in obese individuals, exercise limitation can profoundly restrict daily activities and thus impair quality of life. The American College of Sports Medicine (Jakicic et al., 2001) recommends that obese individuals should exercise at a moderate intensity (55-69% of maximum heart rate), which can be beneficial for the management of their body weight loss. However, maintenance of such exercise intensity may be difficult for them, thus new, innovative protocols have to be established in order to promote the quality of life in obese individuals.

As a prelude to studies investigating the effect of hypoxic exercise on metabolism, as a novel weight management intervention, we initiated a series of studies to examine resting metabolism during 10-day hypoxic confinement. These initial studies were designed to reveal the effect of hypoxia *per se* on metabolism, without any confounding effects of exercise. Interest in this area arises from anecdotal evidence of loss of weight at altitude, or »high altitude anorexia«, and results of controlled studies reporting hypoxia-induced alterations in metabolism (Milledge, 1972; Haufe et al., 2008; Wiesner et al., 2010).

The observation that high altitude exposure leads to considerable weight loss in alpinists (Pugh, 1954; Milledge, 1972), has led to the suggestion that it might be beneficial to incorporate hypoxic training in weight management programs for obese individuals (Mekjavic et al., 2016). It is known that hypoxia stimulates the sympathetic nervous system and field studies have commonly reported weight loss during exposures to high altitude (Gill and Pugh, 1964; Surks et al., 1966; Consolazio et al., 1968; Krzywicki et al., 1969). From these studies, it is not possible to discern whether weight reduction is due to the increased energy expended during hard physical work, non-shivering and shivering thermogenesis in the cold environment, limited availability or palatability of food, dehydration, malabsorption, acute mountain sickness, or a combination of these factors (Boyer and Blume, 1984). Rose et al. (1988) suggested that hypoxia *per se* might be sufficient cause for the weight loss and decreased food consumption reported by mountain expeditions at high altitude. Netzer et al. (2008) reported an average reduction of 1.14 kg in body weight following an 8-wk exercise program conducted under normobaric hypoxic conditions (inspired fraction of O_2_, F_*I*_O_2_ = 0.15) in obese individuals, but the loss of fat mass was not defined. Moreover, loss of appetite was reported after 31-days of hypobaric hypoxic exposure (Westerterp-Plantenga et al., 1999). Similarly, Wasse et al. (2012) reported a suppression of hunger and food intake after only a 7-h exposure to normobaric hypoxia, comprising a 60-min bout of exercise.

Previously (Mekjavic et al., 2016) we reported a significant increase in fat mass during a 10-d normoxic confinement of normal weight individuals, with a tendency, albeit not significant, toward a decrease in fat mass during hypoxic confinement. Adipose tissue is no longer considered as only an energy storage site, but recent studies also emphasize the importance of adipose tissue as a highly active endocrine organ secreting a range of hormones involved in energy metabolism (Meier and Gressner, 2004). Furthermore, gastrointestinal hormones have important roles in energy homeostasis, glucose and lipid metabolism, reproduction, cardiovascular function, and immunity. They directly influence other organ systems, including the brain, liver, and skeletal muscle, and are significantly regulated by nutritional status (Meier and Gressner, 2004). In addition to adiponectin and resistin, adipose tissue also produces leptin—a satiety hormone. Leptin acts on the central nervous system, in particular the hypothalamus, suppressing food intake and stimulating energy expenditure (Webber and Macdonald, 2000).

Our previous study with normal weight individuals demonstrated that a 10-day exposure to normobaric hypoxia without the confounding effect of strenuous physical exercise resulted in decreased food intake (Mekjavic et al., 2016). The aim of the present study was to assess whether hypoxia might initiate similar significant weight loss in men with excess weight, and whether this loss of mass would be predominantly from the fat tissue compartment.

## Materials and Methods

### Study Population

Eight males with excess weight, all low altitude (351.6 ± 103.7 m) residents, participated in two 10-day trials. Inclusion criteria included total body fat>30% and/or body mass index (BMI) > 27.5 kg⋅m^–2^. Their (mean ± SD) age, mass, percent total body fat, and body mass index (BMI) were 30.5 ± 11.1 years, 125.0 ± 17.7 kg, 30.8 ± 6.1%, and 37.6 ± 6.2 kg⋅m^–2^, respectively. Participant exclusion criteria included a history of physician-diagnosed medical problems, recent prolonged exposure to normobaric or hypobaric hypoxia, or recent weight loss, participation in any dietary manipulations in the past 6 months, and use of any medications or drugs. All participants gave their written informed consent to participate in the study. The study protocol was approved by the National Committee for Medical Ethics at the Ministry of Health of the Republic of Slovenia (approval no. 108/08/09) and conformed to the Declaration of Helsinki.

### Experimental Procedure

The study was conducted at the Olympic Sport Centre Planica (Rateče, Slovenia) situated at an altitude of 940 m. We replicated the protocol used previously with normal weight participants (Mekjavic et al., 2016). Participants undertook two trials, during which they were confined to one floor of the facility. In one trial, the ambient conditions were normoxic (NORMOXIA trial), and in the other they were rendered hypoxic (HYPOXIA trial). The study was designed as a randomized cross-over study. Participants were assigned to two groups: initially one group (*N* = 4) was confined to a normobaric normoxic environment (NORMOXIA, F_*I*_O_2_ = 0.2093) for 10 days and the other (*N* = 4) to a controlled normobaric hypoxic environment (HYPOXIA) for the same period. During the HYPOXIA trial, the 10-day exposure to a simulated altitude commenced at 2,800 m (P_*I*_O_2_ = 105 mmHg) on days 1 and 2, and continued with a daily increase of 200 m, until a simulated altitude of 3,400 m (P_*I*_O_2_ = 98 mmHg) was attained. The subjects remained at the simulated altitude of 3,400 m for the remaining 4 days. In both trials, the participants arrived at the facility 3 days prior to the onset of the 10-day confinement. During these 3 days, they were familiarized with the experimental protocol and equipment, and baseline measurements were obtained. Upon completion of the 10-day confinements, participants remained at the facility for an additional 3 days for the post-confinement tests.

After a 3-week wash-out period the participants returned to the Olympic Sport Centre Planica, and the conditions for the participants were crossed-over. During both the NORMOXIA and HYPOXIA trials, participants were confined to the living quarters (each group had at their disposal 3 double sleeping rooms and one living/dining room; total area of ∼ 110 m^2^).

In the HYPOXIA trial, the reduction in FO_2_ was achieved with an oxygen dilution system (B-cat, the Netherlands), based on the Vacuum-Pressure Swing Adsorption principle. The oxygen levels in each room were monitored and recorded at 15-min intervals throughout the 10-day period. In the event that the FO_2_ in any given room decreased below the pre-set value, delivery of the hypoxic gas mixture to that room was stopped. In the event that the FO_2_ dropped by more than 0.005 of the pre-set value, the control system activated a fan, which delivered external ambient air into that room. As a consequence of the fan being activated, the FO_2_ in the room would increase rapidly to the desired level. Once the FO_2_ attained the pre-set value, the fan was de-activated. Moreover, each participant was requested to either wear, or have in close proximity, a personal clip-on type of environmental oxygen analyzer (Rae PGM-1100, California, United States) with an audible alarm that was activated in the event that the oxygen level decreased below the pre-set level.

Prior to the onset of the study, participants completed questionnaires regarding their current and past health status, habitual physical activity levels, dietary habits, and food preferences. During the 3-day period prior to each confinement, participants were requested to record their 3-day dietary intake in a food diary. During the 10-day period, participants’ physical activity was restricted to slow walks in the living area. Nutritional choices and energy intake were documented in daily nutritional diaries during both trials. The food menu comprised typical national foods, freely available, and an effort was made to accommodate individual food preferences. The participants received the same food menu during each 10-day exposure, but without any restriction regarding the quantity consumed; they ate and drank *ad libitum*. The study was conducted at the Olympic Sport Centre Hotel, and the food was prepared by the kitchen staff. Since all meals were prepared according to preset recipes, the meals on a particular day of the study were identical in both normoxic and hypoxic campaigns. The menus were constructed using the application Open Platform for Clinical Nutrition (OPKP)^1^ developed by the Jozef Stefan Institute (Ljubljana, Slovenia). Each day subjects were provided with five meals (breakfast, morning snack, lunch, afternoon snack, dinner). Meals were served at the same time of day. The daily energy intake was recorded and analyzed with the dietary assessment program (OPKP, Jozef Stefan Institute, Ljubljana, Slovenia). Physical activity was monitored continuously with heart-rate (HR) monitors. Participants’ well-being was monitored by medical personnel. All participants had personal pulse oximeters (Nonin, Medicals 3100 WristOx, Minnesota, United States) monitoring capillary oxyhaemoglobin saturation (SpO_2_) and HR. Symptoms of mountain sickness and individual mood and appetite were monitored daily with the Lake Louise Acute Mountain Sickness Score (LLS, Hackett and Oelz, 1992) and Visual Analog Scales (VAS, Sepple and Read, 1989) for Mood and Appetite, respectively. Metabolic measurements were conducted at the same time of day for each subject (COSMED, Quark PFT, Rome, Italy). The metabolic assessments were conducted in a normoxic environment, 1 day prior to the onset of each 10-day confinement (pre-tests), and on day 10 of the confinement period (post-tests) in either hypoxic or normoxic environments.

### Anthropometry

Body composition was analyzed before and after each 10-day confinement with Dual-Emission X-ray absorptiometry (Discovery, Hologic, Inc., Bedford United States). Measurements were made of total body fat mass and regional fat mass (abdominal, right thigh, left thigh) and fat-free mass. Body weight and height were measured with a weighing scale and a stadiometer, respectively (Seca 703, Seca, Hamburg, Germany).

### Metabolic Test

Prior to, and on the final day of each 10-day confinement, participants completed a meal tolerance test (MTT) to assess their fasting metabolic status and the postprandial metabolic responses. For 3 days before the pre-confinement metabolic experiments, participants refrained from any strenuous activity and were limited in their caffeine (daily maximum caffeine content: 150 mg) and alcohol (daily maximum alcohol content: 8 g or 10 ml) consumption. The dietary intake diaries obtained prior to each MTT revealed that the macronutrient composition was similar for the NORMOXIA and HYPOXIA trials. The last meal prior to the MTT was the evening meal the day before the test. For each participant, the composition of the last meal prior to the 12-h fast was the same for each MTT test. For the MTT, participants consumed a standardized mixed nutrient liquid test meal (Ensure, Nutrition shake, vanilla flavor; Abbott) based on their individual body weight (5 ml⋅kg^–1^, 1.5 kcal⋅ml^–1^) determined on the day of the experiment. Additionally, ^13^C-labeled glucose (9.2 mg⋅kg^–1^) was added to the test meal as an indicator of gastric emptying and glucose uptake and use (expired ^13^CO_2_ was measured). The average meal volume was 612.9 ± 100.9 ml and contained 123.8 ± 20.4 g of carbohydrate, 38.3 ± 6.3 g of proteins, and 30.2 ± 4.9 g of fat, plus significant amounts of vitamins and minerals. Participants were asked to consume the entire meal portion provided.

### Blood Sampling

Blood samples (6 ml) were drawn at regular intervals through a catheter (Baxter Health Care, Valencia, CA) inserted into a dorsal hand vein at the beginning of the experiment. The subject’s catheterized hand was placed in a hot box (air temperature 55−60°C) to maintain a constant high hand skin temperature and provide arterialized venous blood. Arterialized venous blood samples were collected in the fasted state prior to the test meal (15 min and approximately 10 s before the meal), and at regular intervals during the 2 h following the ingestion of the test meal, to monitor blood glucose (every 10 min), and to analyze the responses of glucagon-like peptide (GLP-1), serum insulin, peptide YY (PYY) and total ghrelin (every 15 min). Catecholamine (noradrenaline and adrenaline) responses were additionally measured in the fasting state and postprandially. The level of leptin was also measured in the fasted state before and after each trial.

Blood glucose level was analyzed immediately after each arterialized venous blood sample was taken (HemoCue, HMC-201-PROMO, Sweden). For subsequent hormone analyses, all blood samples were either immediately (or after 10 min, in the case of the insulin tube to allow clotting) centrifuged at 3,000 rpm for 10 min (at a temperature of 4°C), and aliquots were kept on ice until the end of the MTT test and stored at −20°C for 1 day, and thereafter at −70°C until further analyses. The analysis of all plasma samples was conducted in duplicate using the assays described below.

Serum insulin concentrations were determined using radio-immunoassay (RIA) (Insulin Coat-A-Count, Diagnostic Products Corp., Los Angeles, United States); GLP-1 concentrations (comprising GLP-1 7-36 amide and 7-37) using Enzyme-Linked ImmunoSorbent Assay (ELISA) (Merck Millipore, Missouri, United States); PYY using RIA for total PYY (Merck Millipore, Missouri, United States) which recognized both PYY1-36 and PYY3-36 in EDTA plasma plus aprotinin; ghrelin was determined using RIA for total ghrelin (Merck Millipore, Missouri, United States) and leptin was determined using a human leptin RIA (Merck Millipore, Missouri, United States). Catecholamines were measured using extraction of the adrenaline and noradrenaline from the plasma (which had been treated with EGTA/glutathione (Sigma-Aldrich, Dorset, United Kingdom) as a preservative) followed by high performance liquid chromatography (HPLC) with electrochemical detection (Forster and Macdonald, 1998).

Labeled glucose artificially enriched with (U-^13^C) glucose (^13^C/C > 99%; Isotec, Miamisburg, OH, United States) was dissolved in each participant’s liquid meal so that the appearance of ^13^CO_2_ in the expired air could be used as a marker of the combination of gastric emptying, uptake and oxidation of the test meal. This breath testing is based on the principle that an ingested substrate is metabolized, and a measurable metabolite is then expelled by the respiratory system. Therefore, breath samples were collected simultaneously with the blood samples before and after the meal (at -15, 0, 15, 30, 45, 60, 75, 90, 105, and 120 min) using a breath-sampling bag (500 ml) with a one-way valve for capture of normal exhaled air. To allow analysis of ^13^C/^12^C in expired CO_2_, 20 ml samples of expired gasses were collected from the breath-sampling bag via a catheter (Baxter Health Care, Valencia, CA) into evacuated tubes (Vacutainers; Becton Dickinson, Franklin Lakes, NJ) and stored until further analysis. All samples were subsequently analyzed in triplicate with mass spectrometry (Prism, VG, Manchester, United Kingdom) and ^13^C abundance was calculated for all time points. The incremental postprandial response (i.e., the ^13^C enrichment curve) was integrated and presented as an area under the curve (AUC).

#### Assessment of Metabolic Rate During Rest

One hour after participants completed their morning personal hygiene and morning body weighing, they relaxed and rested supine for 3 h during the metabolic test. The metabolic tests were conducted at the same time of the day for each participant (between 07:00 a.m. and 01:00 p.m.); the environmental conditions were kept constant: the mean ambient temperature, relative humidity and barometric pressure were 24 ± 0.8^°^C, 37.4 ± 4.6% and 682 ± 4 mmHg, respectively. The conditions of the laboratory were thermally comfortable. Resting energy expenditure (REE) was measured using indirect respiratory calorimetry with an open-circuit spirometer canopy system (Quark RMR, Cosmed, Italy). A transparent ventilated hood system was placed over the participant’s head with a hose connecting the hood to the gas analysis system. The flow of ambient air through the hood was controlled by a pump. The analyzer was calibrated in a normoxic environment for all the tests. Reference gas standards were used to calibrate the system, and all measurements were automatically corrected for environmental temperature, pressure, and humidity. The post-tests in the HYPOXIA trial were performed in a hypoxic environment. During the hypoxic post-tests the metabolic analyzer was calibrated in a normoxic environment, but fasting and post-prandial REE were monitored in the hypoxic environment of the room. Since the hardware and software of the metabolic analyzer (Quark RMR, Cosmed, Italy) was not capable of conducting on-line calculations of REE in a normobaric hypoxic environment, the values of REE in the hypoxic environment were derived manually from the measurements of the FO_2_ entering and exiting the canopy placed on the subject’s head, and the air flow through the canopy. Basal measurements of $\dot{V}$O_2_ and CO_2_ production ($\dot{V}$CO_2_) were conducted at 15-min intervals with the individuals awake, before the standard meal and at time-points 15, 45, 75, 105 min, postprandially. To disregard the artifacts that occur during the first few minutes of measurement, when the canopy is placed over a subject’s head and the measurement initiated, the calculation of REE was made on the values measured after the first 2 min of the test.

Additionally, HR (Polar, RS400, Finland), arterial pressure (aneroid sphygmomanometer, Welch Allyn, Inc., United States), SpO_2_ (Nonin Medicals 3100 WristOx, Minnesota, United States) were recorded at 15-min intervals.

#### Appetite Sensation

Subjective perceptions of hunger, fullness, desire to eat, thirst and prospective food consumption were assessed using a validated visual analog scale (VAS; Stubbs et al., 2000). The VAS scale was a 100 mm line, anchored to the left with “sensation not felt at all” and to the right with “sensation felt the greatest” and subjects were asked to place a vertical line in relation to their feeling at that particular point in time, these scores were then summed to form the Composite Satiety Score (CSS) according to the following equation (the higher the score, the higher the level of subjective satiety).


CSS=[Full+(100-Desire)+(100-Hunger)+(100-PFC)]/4

where,

PFC = prospective food consumption.

### Intestinal Blood Flow

Blood flow was measured in the proximal portion of the superior mesenteric artery (SMA), 2–5 cm distally to its origin in the aorta. Flow was estimated by measurements of vessel-lumen diameter and mean flow-velocity, using ultrasonographic/Doppler techniques (Philips CX50, Bothel, Washington). Flow velocity was measured using a 3–12 MHz linear array Doppler transducer that was kept aligned with the vessel; the angle correction of the transducer was maintained at less than 60° and its sample volume was adjusted to cover more than 75 % of the vessel lumen. The diameter of the SMA was measured in B-mode image during end-diastole (determined from the ECG), as wall-to-wall distance in the sagittal section. Assuming that the artery had a circular cross-section, flow was subsequently calculated by multiplying vessel cross-sectional area by the mean flow-velocity. Each flow determination was the average of measurements from 3 to 4 consecutive heart beats, repeated 2–3 times, i.e., the average of data from 6 to 12 beats. The same sonographer performed all measurements.

### Calculations and Statistical Methods

Mass spectrometer analysis of breath samples provided a delta value (δ), which was used to calculate abundance (atom %) at each time point using standard methods (Pouteau et al., 1998). Enrichment of breath samples was then obtained by standardizing postprandial values to those at baseline.

The homeostatic model assessment (HOMA) method was used to quantify insulin resistance and β-cell function (Matthews et al., 1985). The non-linear model of two types of HOMA scores were used:

**HOMA IR** = insulin resistance = (fasting insulin in mU⋅l^–1^) × (fasting plasma glucose in mmol⋅l^–1^) / 22.5
**HOMA β** = β-cell function [%] = 20 × (fasting insulin in mU⋅l^–1^) / [(fasting glucose in mmol⋅l^–1^)—3.5]

A 2-way ANOVA (NORMOXIA-HYPOXIA, Pre-Post) with repeated measures was used to define the effect of the 10-day confinements on the measured variables. A Tukey *post hoc* test was used to assign the specific differences in the analysis of variance. Additionally, some data were presented as calculated area under curve (AUC) and compared with the same type of ANOVA. Values are mean ± SD unless indicated otherwise. The significance level was set at 0.05.

## Results

**FIGURE 1:**
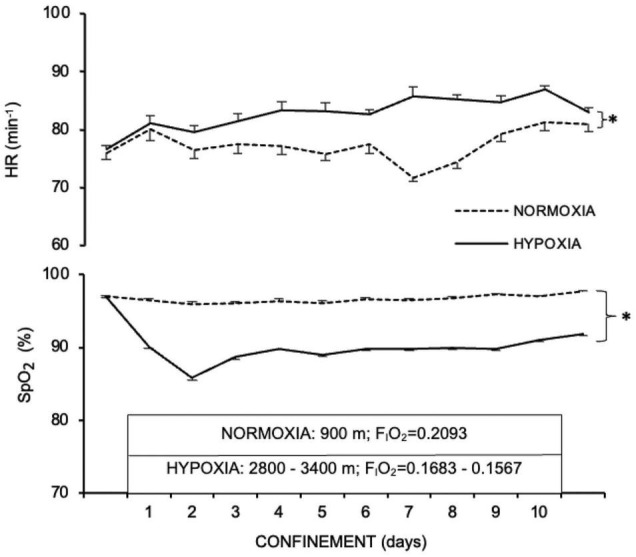
Nocturnal heart rate (HR, min^– 1^) and capillary oxyhaemoglobin saturation (SpO_2,_ %) before (PRE), during, and at the end (POST) of the NORMOXIA and HYPOXIA trials. Values are mean ± SEM, *n* = 8. ^∗^Significant differences between NORMOXIC and HYPOXIC confinement; *p* < 0.05.

The average nocturnal capillary oxyhaemoglobin saturation (SpO_2_) during the course of the 10-day confinement was lower (*p* < 0.001) in HYPOXIA (90.3 ± 0.2%) than in NORMOXIA (97.4 ± 0.1 %). In contrast, heart rate (HR) was higher (*p* = 0.04) in HYPOXIA (82.7 ± 0.8 min^–1^) than NORMOXIA (78.8 ± 1.0 min^–1^, Figure 1). Furthermore, arterial pressures were similar before and after both the NORMOXIC (Pre: systolic arterial pressure, SAP = 144 ± 15 mmHg, diastolic arterial pressure, DAP = 88 ± 10 mmHg; Post: SAP = 138 ± 18 mmHg, DAP = 87 ± 12 mmHg), and HYPOXIC (Pre: SAP = 145 ± 14 mmHg, DAP = 89 ± 11 mmHg; Post: SAP = 137 ± 9 mmHg, DAP = 87 ± 10 mmHg) confinements. No symptoms of mountain sickness (Lake Louise Score) were detected during the HYPOXIC confinement.

The changes in body weight were different (*p* = 0.02; Table 1) between the two 10-day confinements. In particular, body weight decreased by 0.7 ± 0.2 kg in HYPOXIA, whereas it increased by 1.0 ± 0.2 kg in NORMOXIA; a difference of 1.7 kg between the two interventions. Neither the HYPOXIC nor the NORMOXIC confinement affected the total or regional body fat mass (Table 1).

**TABLE 1:** Anthropometric variables before (PRE), and at the end (POST) of the 10-day NORMOXIC and HYPOXIC trials.

	Normoxia Pre	Post	Hypoxia Pre	Post
Body weight (kg)	125.0 ± 17.7	126.0 ± 19.3	123.9 ± 18.0	123.1 ± 19.0
BMI (kg.m^–2^)	37.6 ± 6.2	37.9 ± 6.7	37.3 ± 6.3	37.1 ± 6.6
Lean mass (kg)	77.2 ± 6.9	76.9 ± 8.3	75.7 ± 7.5	74.5 ± 7.1
Total fat mass (%)	30.8 ± 6.1	31.6 ± 5.3	32.1 ± 6.7	31.8 ± 6.1
Abdominal fat (%)	35.1 ± 5.4	36.0 ± 5.2	36.5 ± 5.7	36.2 ± 6.0
Right thigh fat (%)	29.0 ± 6.3	30.3 ± 5.5	30.0 ± 5.9	30.6 ± 6.2
Left thigh fat (%)	28.1 ± 6.3	29.9 ± 5.7	28.4 ± 5.8	30.9 ± 8.0

*Values are mean ± SD.*

**FIGURE 2:**
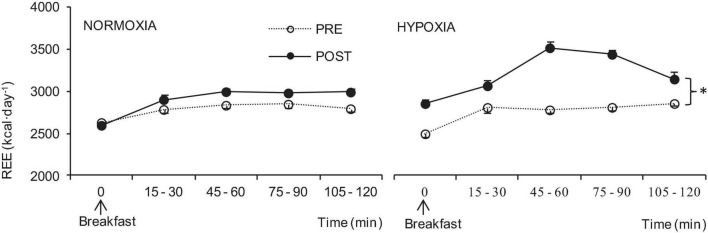
Postprandial resting energy expenditure (REE) before (PRE), and at the end (POST) of the NORMOXIA and HYPOXIA trials. Values are mean ± SEM, *n* = 8. ^∗^Significant differences between PRE and POST confinement; *p* < 0.05.

REE in the fasted state was elevated after HYPOXIC confinement (358 ± 49 kcal⋅day^–1^, *p* = 0.03); after NORMOXIC confinement fasting REE remained at similar levels (-33 ± 18 kcal⋅day^–1^). Furthermore, after HYPOXIC confinement postprandial REE increased (*p* = 0.05) while no differences were observed after NORMOXIC confinement (Figure 2).

The mean energy intake was not significantly different during the NORMOXIC and HYPOXIC confinements, although there was a tendency for it to be lower in HYPOXIA (p = 0.08). Specifically, energy intake was 3,497 ± 189 kcal⋅day^–1^ in NORMOXIA and 3,264 ± 164 kcal⋅day^–1^ in HYPOXIA.

The hormone and metabolite concentrations before and after the NORMOXIA and HYPOXIA confinements in the fasted and postprandial states are presented in Table 2. The hemoglobin level was unaffected by the HYPOXIC confinement, whereas it decreased after the NORMOXIC confinement. Cholesterol, triglycerides high-density lipoprotein (HDL) and low-density lipoprotein (LDL) levels were unaffected by both confinements. Fasting PYY was significantly decreased both by the NORMOXIC and HYPOXIC confinement, whereas there was a tendency of GLP-1 to be higher after than before each confinement. In the fasted state, all other blood variables remained unaltered by the NORMOXIC as well as the HYPOXIC confinement.

**TABLE 2:** Hematological variables in fasted state after the meal before (PRE) and at the end (POST) of the NORMOXIC and HYPOXIC trials.

Hematological variables	Normoxia				Hypoxia			
	Pre		Post		Pre		Post	
	Fasted	Postprandial state	Fasted	Postprandial state	Fasted	Postprandial state	Fasted	Postprandial state
Hemoglobin (g⋅dL^–1^)	154.1 ± 12.3	−	147.6 ± 13.8^†^	−	154.8 ± 13.6	−	156.0 ± 11.2*	−
Cholesterol (mg⋅L^–1^)	4.9 ± 1.1	−	4.4 ± 1.1	−	4.9 ± 1.1	−	4.2 ± 1.0	−
Triglyceride (mg⋅dL^–1^)	2.3 ± 1.4	−	1.9 ± 1.4	−	2.4 ± 1.4	−	2.1 ± 1.3	−
HDL (mg⋅dL^–1^)	1.1 ± 0.6	−	0.9 ± 0.3	−	1.1 ± 0.7	−	0.9 ± 0.3	−
LDL (mg⋅dl^–1^)	2.9 ± 0.7	−	2.7 ± 0.7	−	2.9 ± 0.7	−	2.5 ± 0.7	−
Blood glucose (mmol⋅L^–1^)	4.7 ± 0.5	7.5 ± 0.6^#^	4.7 ± 0.7	7.5 ± 0.7#	4.4 ± 0.6	7.1 ± 0.8^#^	4.4 ± 0.5	7.7 ± 0.7#*
Insulin (pmol⋅L^–1^)	19.0 ± 18.9	150.3 ± 61.7^#^	17.6 ± 13.8^†^	195.1 60.3^#^	14.6 ± 11.0	171.7 ± 54.1^#^	15.5 ± 10.8	188.5 ± 42.3^#^
HOMA-IR	0.7 ± 0.8	−	0.6 ± 0.5	−	0.5 ± 0.4	−	0.6 ± 0.4	−
GLP-1 (pM)	2.1 ± 1.0	3.2 ± 1.3^#^	2.4 ± 1.6	3.2 ± 1.2^#^	1.9 ± 0.9	3.4 ± 2.2^#^	2.2 ± 0.9	3.3 ± 1.2^#^
Adrenaline (nmol⋅L^–1^)	0.2 ± 0.1	0.1 ± 0.1^#^	0.2 ± 0.1	0.1 ± 0.1#	0.2 ± 0.1	0.1 ± 0.1#	0.1 ± 0.1	0.1 ± 0.1#
Noradrenaline (nmol⋅L^–1^)	1.2 ± 0.3	1.5 ± 0.5	1.3 ± 0.7	1.4 ± 0.6	1.1 ± 0.3	1.3 ± 0.2	1.3 ± 0.5	1.5 ± 0.4
Ghrelin (pg⋅ml^–1^)	925.6 ± 333.4	833.1 ± 245.1^#^	945.8 ± 367.8	826.5 ± 278.3^#^	965.1 ± 394.2	873.2 ± 273.4^#^	940.0 ± 216.9	840.4 ± 176.0^#^
Leptin (ng⋅ml^–1^)	18.6 ± 9.4	−	21.1 ± 11.6	−	17.1 ± 10.0	−	18.5 ± 9.9	−
PYY (pg⋅ml^–1^)	105.9 ± 40.3	136.1 ± 36.2^#^	97.2 ± 24.8^†^	129.9 ± 25.7^#^	103.0 ± 22.7	137.4 ± 49.6^#^	91.9 ± 17.8^†^	129.8 ± 48.2^#^

*Values are mean ± SD.*

*HDL, high-density lipoprotein; LDL, low-density lipoprotein; HOMA-IR, homeostasis model assessment–insulin resistance, GLP-1, glucagon-like peptide-1; PYY, peptide YY_3–36_.*

^†^significant (p ≤ 0.05) differences between NORMOXIA and HYPOXIA in the same state; *significant (p ≤ 0.05) differences between pre and post in the same confinement.

**FIGURE 3:**
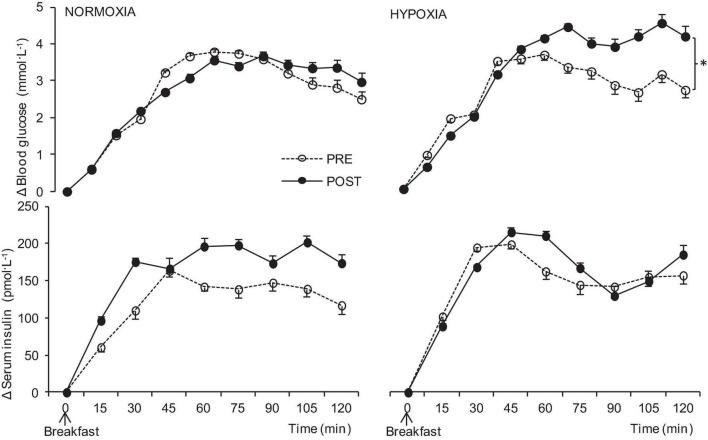
Postprandial delta blood glucose and serum insulin before (PRE), and at the end (POST) of the 10-day normoxic (NORMOXIA) and hypoxic (HYPOXIA) confinements. Values are mean ± SEM, *n* = 8. ^∗^Significant differences between PRE and POST confinement; *p* < 0.05.

The blood glucose level increased after each MTT. The postprandial blood glucose response was higher after the HYPOXIA confinement than after the NORMOXIA confinement (*p* < 0.001, Figure 3). Insulin levels also increased after the test meals, but no significant increases in insulin in the post-HYPOXIC confinement were observed (Figure 3). No significant differences (*p* = 0.8) were observed in the postprandial GLP-1 increases in either NORMOXIA or HYPOXIA. Moreover, there were no significant changes in postprandial adrenaline level, while noradrenaline levels increased (*p* < 0.001) after the meal in both the NORMOXIC and HYPOXIC trials. Postprandial total ghrelin levels decreased after the meal (Table 2, *p* < 0.001), there being no difference between the NORMOXIA and HYPOXIA (*p* = 0.16). PYY values (Table 2) were increased after the meal (*p* < 0.001), with higher responses after the HYPOXIC confinement (*p* = 0.01).

**FIGURE 4:**
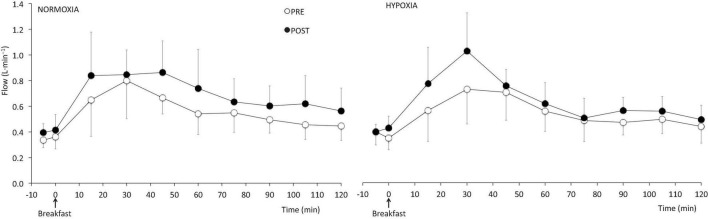
Postprandial blood flow of the superior mesenteric artery before (PRE) and at the end (POST) of the 10-day normoxic (NORMOXIA) and hypoxic (HYPOXIA) confinements. Values are mean ± SEM, *n* = 8.

The increments in flow and diameter of the SMA in response to the MTT were similar before and after both the NORMOXIC and HYPOXIC confinements (Figure 4).

Moreover, there were no differences in subjective satiety, presented as CSS, in the fasted state before compared to after NORMOXIC or HYPOXIC confinement. Similarly, there were no differences (*p* = 0.51) postprandially in subjective satiety before compared to after the NORMOXIC or HYPOXIC confinement.

## Discussion

The main observation of the present study is that of a significant loss in body mass in men with excess weight after a continuous 10-day normobaric hypoxic confinement, compared to an increase in body mass during a normoxic confinement of similar duration. Previous observations of high altitude anorexia in climbers may have been confounded by increased physical activity, insufficient food availability and environmental cold. The present study confirms that hypoxia may lead to body weight reduction in the absence of these cofounders, predominantly due to an increase in REE.

### High Altitude Anorexia

Our observation is in agreement with many field studies that have reported remarkable weight loss at altitudes higher than 4,000 m (Westerterp et al., 1992, 1994; Pulfrey and Jones, 1996; Reynolds et al., 1999; Westerterp, 2001; Louis and Punjabi, 2009; Kayser and Verges, 2013; Wandrag et al., 2017). In such field studies, it is difficult to exclude the contribution of cold, physical activity, food availability and palatability, commonly present at high altitude, from the effects of hypoxia *per se*. In the present study, we investigated the responses to 10-days of continuous hypoxia. Gunga et al. (2003) investigated the effect of 3 weeks at moderate altitude (1,700 m) in male subjects with metabolic syndrome (age: 54 years; BMI: 30.3 kg.m^–2^; with hypertension, hyperlipidemia, diabetes mellitus and/or coronary heart disease). They reported a body weight decrease of 0.9 kg at day 19 at altitude, and a further reduction of 0.7 kg in body weight 6–7 weeks after the altitude exposure. This weight loss was due to a reduction in body fat, whereas no change was observed in lean body mass. However, during the altitude exposure the subjects with metabolic syndrome were active, participating in activities such as mountain hiking between 1,500 and 2,500 m and swimming. Therefore, and because there was no control group, it is not possible to conclude the extent to which the hypoxia *per se* or the physical activity contributed to the weight change. In addition, in the study of Gunga et al. (2003) energy intake was analyzed retrospectively from the questionnaires filled out by participants and from this an average total energy intake of approximately 1,900 kcal⋅day^–1^ was derived; whilst REE was not measured. This value of 1,900 kcal⋅day^–1^ was likely a substantial underestimation, as the subjects weight of 92.9 kg would require at least 2,222 kcal⋅day^–1^ to maintain body weight with a low level of physical activity (derived using the Harris-Benedict equation, revised by Roza and Shizgal, 1984), and substantially more if the level of physical activity was higher. Alternatively, the estimate of a 1,900 kcal⋅day^–1^ energy intake was correct, in which case the body weight loss is attributable to low energy intake rather than a hypoxia-induced change in metabolism. Similarly, Lippl et al. (2010) observed body weight loss in obese individuals after 14 days at altitude, and concluded that, whatever the cause, it seemed clear that increases in REE contributed to the weight loss. However, in the absence of any control groups or control trials in the aforementioned studies, it is not possible to conclude the extent to which either the hypoxia *per se* or the physical activity contributed to the weight change.

While there were some early indications of unchanged REE at altitude (Stickney and Van Liere, 1953; Chiodi, 1957; Durnin and Brockway, 1959), many investigators have observed an elevated REE at altitude, most likely due to an increased activity-induced energy expenditure in climbing subjects (Picon-Reategui, 1961; Grover, 1963; Gill and Pugh, 1964; Klausen, 1966; Hannon and Sudman, 1973; Butterfield et al., 1992; Pulfrey and Jones, 1996). In most field studies metabolic activity was measured before and after the expeditions, either near to sea level, or from average daily metabolic activity determined from the activity level in the field, and the resting metabolic rate for climbers at altitude was assumed to be the same as at sea level (Westerterp et al., 1992, 1994). However, high altitude anorexia was reported also in hypobaric laboratory studies (Westerterp et al., 2000) and there are also indications of similar effects of normobaric hypoxia. Netzer et al. (2008) reported significant body weight loss after normobaric hypoxic training, but the mechanism of this weight loss remains unresolved. To our knowledge there are as yet no data regarding REE levels during prolonged normobaric hypoxic confinement in the absence of changes in environmental temperature, physical activity and diet. Matu et al. (2017) reported a significantly higher REE during a 5-h exposure to a simulated altitude (normobaric hypoxia) of 4,300 (2,242 ± 269 kJ) compared to sea level (1,826 ± 230 kJ) and a simulated altitude of 2,150 m (1,924 ± 217 kJ). The difference in REE between sea level and 2,150 m simulated altitude was not significant. In the present study, the significantly higher metabolic needs (elevated REE values) after 10-day normobaric HYPOXIA in men with excess weight could be responsible for the observed body weight loss. Despite similar (but not identical) effects of hypobaric and normobaric hypoxic exposures (Richard and Koehle, 2012), the ventilatory response appears to be even more pronounced in normobaric hypoxia (Evetts et al., 2005). In part, the energy cost of such hyperpnea could explain the observed higher REE in HYPOXIA. Our observation is in agreement with studies reporting a 15–26% elevation of REE in patients with chronic obstructive pulmonary diseases (COPD; Vasconcelos et al., 2002; Sergi et al., 2006). Recently, Ramires et al. (2012) have suggested that the increased REE in COPD patients is due to hypermetabolism, greater respiratory muscle effort, higher O_2_ requirements and inflammation.

### Insulin Sensitivity

The endocrine factors that may have contributed to the observed changes in body weight remain unclear. Although improved short-term glycemic control has been reported after acute hypoxia and after hypoxia combined with exercise (Mackenzie et al., 2011), we observed significantly increased glucose levels after the HYPOXIA trial. The mechanism could be a tendency for insulin resistance. Markedly reduced insulin sensitivity in healthy men and women at high altitude has been already attributed to the hypoxic stimulus (Larsen et al., 1997; Braun et al., 2001). In addition, similar responses have been observed in lean and obese mice by Reinke et al. (2011), who reported insulin resistance concomitant with significant increases in leptin, and severe systemic inflammation following two hypoxic regimens (intermittent and sustained hypoxia).

Moreover, the present subjects had excess weight and adipose tissue is recognized as a source of pro-inflammatory cytokines that are linked to insulin resistance in muscle and liver (Guzik et al., 2006). There are some suggestions that this pro-inflammatory state is due, in part, to chronic hypoxia induced in the adipose tissue of obese individuals, giving rise to a cascade of biochemical events leading to the release of the proinflammatory/insulin resistance mediating cytokines (Trayhurn and Wood, 2004). Thus, it is possible that normobaric hypoxia may exacerbate this effect of the cytokines, which may lead to increased insulin resistance, but further research is needed to evaluate this.

### Appetite Hormones

Body weight reduction may be a result of an imbalance in the communication between the gastrointestinal tract and nervous system required for gut-brain signaling of the food intake control (Konturek et al., 2004; Hussain and Bloom, 2013). Moreover, disturbed body weight regulation at high altitude can be caused by appetite change or altered requirements, or a combination of both. In the present study there were no significant changes in energy intake after the NORMOXIA trial, but a tendency toward decreased intake after the HYPOXIA trial.

Present observations are in agreement with those of Westerterp et al. (1992, 1994) who reported decreased body weight mainly due to decreased appetite and an associated decrease of caloric intake at simulated high altitude (hypobaric hypoxia). It has been proposed that gut hormones may be a useful target for anti-obesity therapy (Murphy and Bloom, 2004; Cummings and Overduin, 2007; Hussain and Bloom, 2013). For example, PYY is thought to have a critical role in energy intake inhibition (Batterham et al., 2002). PYY is released into the circulation proportional to food intake within 1-h post-feeding (Adrian et al., 1985). Wasse et al. (2012) investigated the acute effects of normobaric hypoxia on PYY and acylated ghrelin (appetite stimulator). They reported a suppression of energy intake after 7 h of normobaric hypoxia (F_*I*_O_2_ = 12.7, simulated altitude 4,000 m), a suppression of acylated ghrelin concentrations and a tendency for suppressed PYY. Although we observed the anticipated decrease in total ghrelin postprandially, there were no differences in its level and response between the 10-day NORMOXIA and HYPOXIA trials, thus not lending support to an exacerbated ghrelin-induced suppression of appetite during hypoxic exposure, as suggested by Wasse et al. (2012). Our finding of an increase in postprandial PYY after prolonged HYPOXIA concomitant with a reduction of spontaneous energy intake could support the notion of a potentially beneficial role of hypoxia in obesity prevention.

Leptin levels provide feedback regarding adipose tissue mass to central regions and regulate food intake. Although the observed levels of leptin in the subjects participating in the present study are in agreement with the higher levels normally observed in obese individuals (Rosická et al., 2003) compared to non-obese, there were no significant changes in leptin levels as a consequence of either the NORMOXIC or HYPOXIC confinement.

### Mesenteric Artery Flow

A suppressed total ghrelin level after 7 h of normobaric hypoxia has been associated with impaired gut blood flow at high altitude (Wasse et al., 2012). Namely, a decreased flow in the SMA after acute hypobaric hypoxia (2 h, equivalent to 4,800 m) compared with flow in normoxia was reported earlier by Loshbaugh et al. (2006). They hypothesized that, if reduction in blood flow was maintained during prolonged exposure, it might be responsible for reduced appetite and weight loss at altitude. In contrast, Kalson et al. (2010) reported decreased energy intake and increased resting blood flow in the gastrointestinal tract during acute exposure to high altitude hypoxia (2 nights acclimatization at 3,300 m; measurements were made 24 h after exposure to 4,329 m). Furthermore, in their study the increased mesenteric artery flow response following food ingestion was maintained. They concluded that reduced blood flow is unlikely to cause gastrointestinal responses (increase in gut hormones) and reduced appetite at high altitude. However, the proposed vascular mechanism of potential changes in acylated ghrelin or PYY (Wasse et al., 2012) during longer exposure to hypoxia was not clear. Our observations of unchanged flow response in the mesenteric artery after 10-day HYPOXIA measured in hypoxic conditions supports the suggestion of Kalson et al. (2010), that impaired gut blood flow does not contribute to high altitude anorexia after several days. Further research is needed regarding neurohormonal and vascular satiety signaling, which in the present study did not reach significant differences, but may nevertheless contribute to reduced appetite.

### Weight Loss

The present study is in agreement with those suggesting that controlled hypoxia might be incorporated in weight loss protocols (Netzer et al., 2008; Wasse et al., 2012). Previous altitude studies have indicated that the decrease in weight is mainly attributable to a loss of fat (Rose et al., 1988; Armellini et al., 1997; Westerterp-Plantenga et al., 1999; Gunga et al., 2003). For example, Fusch et al. (1996) reported that 70% of the weight loss after 2 months at high altitude (4,900 m) was due to body fat loss. Similarly, Boyer and Blume (1984) reported that 33% of the weight loss after 10 days at 6,000 m was due to loss of fat content. Ge et al. (2010) reported reduced waist circumference after a month at an altitude of 4,638 m. In addition, the degree of weight loss positively correlated with the baseline body weight, suggesting that more body weight will be lost during a stay at high altitude, if the initial body weight is higher. In our study, the changes in body composition were too small to define whether body weight loss was due to loss of fat or lean body mass (Table 1), or whether, and to what extent dehydration contributed to the weight loss. Similarly, the previously reported loss of body weight after normobaric hypoxic training in obese individuals (Netzer et al., 2008) was not defined as regards the share of the loss being contributed by fat, lean body and water. Specifically, Netzer et al. (2008) reported alterations in fat metabolism. Namely, there was a tendency for triglycerides, low density lipoproteines (LDL), cholesterol to be reduced while high density cholesterol (HDL) remained stable, however, as in our study, none of these changes reached the level of statistical significance (Table 2).

## Summary

A limitation of the present study is the small sample size. Nevertheless, the results point to some potential benefits of normobaric hypoxic confinement, which warrant further investigation. Normobaric hypoxic confinement (HYPOXIA) in the present study resulted in significantly increased levels of REE, suggesting a potential value of hypoxia in achieving weight loss in obese individuals. The observed tendency for decreased energy intake during hypoxia combined with the enhancement of PYY secretion under hypoxic conditions may be one of the potential mechanisms for the hypoxia induced body weight loss (Karra et al., 2009; Hussain and Bloom, 2013). An optimal weight loss regimen should also involve physical activity to control glucose regulation, especially if it is compromised by hypoxia, but this needs to be explored further.

## Data Availability Statement

The raw data supporting the conclusions of this article will be made available by the authors, without undue reservation.

## Ethics Statement

The studies involving human participants were reviewed and approved by the Komisija za medicinsko etiko, Ministrstvo za zdravje. The patients/participants provided their written informed consent to participate in this study.

## Author Contributions

IBM, OE, and IAM conceived and designed the study. Together with MA, ES, and RK conducted the studies, analyzed, and interpreted the results. IBM and MA drafted the manuscript. ES, RK, OE, and IAM critically revised the manuscript. All authors approved the final version of the manuscript and agreed to be accountable for all aspects of the work in ensuring that questions related to the accuracy or integrity of any part of the work are appropriately investigated and resolved. All persons designated as authors qualify for authorship, and all those who qualify for authorship are listed.

## Conflict of Interest

The authors declare that the research was conducted in the absence of any commercial or financial relationships that could be construed as a potential conflict of interest.

## Publisher’s Note

All claims expressed in this article are solely those of the authors and do not necessarily represent those of their affiliated organizations, or those of the publisher, the editors and the reviewers. Any product that may be evaluated in this article, or claim that may be made by its manufacturer, is not guaranteed or endorsed by the publisher.

## Footnotes

1
www.oplp.wi

## Abbreviations

BMI: Body mass index
CSS: Composite satiety score
ECG: Electrocardiogram
ELISA: Enzyme-linked immunosorbent assay
EPO: Erythropoietin
F_*i*_O_2_: Fraction of inspired oxygen
HOMA: Homeostatic food consumption
HOMA IR: Insulin resistance
HOMA ß: cell function
HR: Heart rate
MTT: Meal tolerance test
PFC: Prospective food consumption
P_*i*_O_2_: Partial pressure of inspired oxygen
PYY: Peptide YY
REE: Resting energy expenditure
RH: Relative humidity
RIA: Radio-immunoassay
S_*p*_O_2_: Capillary oxyhaemoglobin saturation
VAS: Visual analog scale
$\dot{V}$O_2_: Oxygen uptake
$\dot{V}$CO_2_: Carbon dioxide production.
